# Liquid-Liquid Phase Separation in the Prognosis of Lung Adenocarcinoma: An Integrated Analysis

**DOI:** 10.2174/0115680096345676241001081051

**Published:** 2024-11-06

**Authors:** Qilong Wang, Nannan Sun, Jianhao Li, Fengxiang Huang, Zhao Zhang

**Affiliations:** 1 Department of Respiration, The First Affiliated Hospital of Zhengzhou University, Zhengzhou, Henan, People's Republic of China;; 2 Department of Hematology, The First Affiliated Hospital of Zhengzhou University, Zhengzhou, Henan, People's Republic of China;; 3 Precision Medicine Center, The First Affiliated Hospital of Zhengzhou University, Zhengzhou, Henan, People's Republic of China

**Keywords:** Lung adenocarcinoma, liquid-liquid phase separation, KRT6A, prognosis, EGFR, IGF2BP1

## Abstract

**Background:**

Lung adenocarcinoma (LUAD) is a highly lethal malignancy. Liquid-Liquid Phase Separation (LLPS) plays a crucial role in targeted therapies for lung cancer and in the progression of lung squamous cell carcinoma. However, the role of LLPS in the progression and prognosis of LUAD remains insufficiently explored.

**Methods:**

This study employed a multi-step approach to identify LLPS prognosis-related genes in LUAD. First, differential analysis, univariate Cox regression analysis, Random Survival Forest (RSF) method, and Least Absolute Shrinkage and Selection Operator (LASSO) Cox regression analysis were utilized to identify five LLPS prognosis-related genes. Subsequently, LASSO Cox regression was performed to establish a prognostic score termed the LLPS-related prognosis score (LPRS). Comprehensive analyses were then conducted based on the LPRS, including survival analysis, clinical feature analysis, functional enrichment analysis, and tumor microenvironment assessment. The LPRS was integrated with additional clinicopathological factors to develop a prognostic nomogram for LUAD patients. Immunohistochemical validation was performed on clinical tissue samples to further validate the findings. Finally, the relationship between KRT6A, one of the identified genes, and epidermal growth factor receptor (EGFR) mutations was investigated.

**Results:**

The LPRS was established using five LLPS-related genes: IGF2BP1, KRT6A, LDHA, PKP2, and PLK1. Higher LPRS was closely associated with poor survival outcomes, gender, progression-free survival (PFS), and advanced TNM stage. Furthermore, LPRS emerged as an independent prognostic factor for LUAD. A nomogram integrating LPRS, TNM stage, and age demonstrated remarkable predictive accuracy for prognosis among patients with LUAD. LLPS likely influences LUAD prognosis through the activity of IGF2BP1, KRT6A, LDHA, PKP2, and PLK1. KRT6A exhibits significant upregulation in LUAD, particularly in patients with EGFR mutations.

**Conclusion:**

This study introduces a novel LPRS model that demonstrates high accuracy in predicting the clinical prognosis of LUAD. Moreover, the findings suggest that KRT6A may play a critical role in the LLPS-mediated malignant progression of LUAD.

## 1 INTRODUCTION

Lung cancer is a leading form of cancer, ranking highest in global cancer mortality rates [1, 2]. It was projected that the incidence of lung cancer exceeded 2.2 million cases in 2020. Non-Small Cell Lung Cancer (NSCLC) accounts for over 85% of all cases and can be categorized by histological subtype [3, 4]. Adenocarcinoma is the most common type of NSCLC globally, accounting for 40% of cases, with squamous cell carcinoma following at 25% [5]. The progress in gene detection and targeted therapy has greatly improved the early detection and treatment outcomes for lung adenocarcinoma (LUAD) [6-10]. Over the past decade, the introduction of epidermal growth factor receptor (EGFR) targeted therapy has transformed the treatment of LUAD, particularly for patients with advanced-stage disease who have a targetable driver alteration [11, 12]. Despite significant advancements in the management and support of LUAD, the five-year survival rate remains suboptimal [13-16]. EGFR mutations promote the proliferation and metastasis of LUAD cells while also providing better therapeutic targets; however, EGFR resistance remains a significant contributor to poor patient prognosis [17-19]. Consequently, novel and effective biomarkers are necessary to evaluate prognosis and predict treatment responses, thereby assisting patients with LUAD in making clinical decisions.

Liquid-liquid phase separation (LLPS) is a biophysical process in which biomolecules rapidly and transiently assemble to create liquid-like condensates. This phenomenon facilitates the formation of membrane-less organelles within cells, contributing to the organization of cellular compartments [20, 21]. These compartments and organelles are essential for various cellular functions, including chromatin reorganization, gene transcription regulation, and translation [22, 23]. LLPS dynamics involve the active participation of scaffolds, regulators, and client molecules. Scaffolds provide structural integrity, regulators ensure functional efficiency, and client molecules frequently exhibit specific binding to scaffold components [21]. The role of LLPS in regulating the biological activities and signaling pathways of essential proteins, such as RNA-binding proteins and transcription factors, is widely recognized [24, 25]. LLPS plays a pivotal role in the formation of biomolecular condensates through the RNA-binding protein YTHDC1, which safeguards specific mRNAs from degradation [26]. Recent studies highlight the connection between LLPS and various diseases, particularly neurodegenerative disorders, such as ALS and Alzheimer's disease [27-31]. Research has demonstrated the critical significance of LLPS in lung cancer treatment, including the identification of cancer-promoting proteins and the development of innovative LLPS-based drug delivery systems [30, 32-35]. Thus, investigating the impact of LLPS in LUAD studies is considered a promising approach to increase our understanding of the disease mechanisms, improve predictions of disease progression, and personalize treatment strategies.

This study developed a prognostic signature for LUAD and identified potential therapeutic compounds for patients with a high liquid-liquid phase separation-related risk score (LPRS). Concurrently, screenings and experimental validations were performed, revealing that keratin 6A (KRT6A) is significantly overexpressed in LUAD, and its overexpression is closely associated with advanced TNM staging and EGFR mutations. The LPRS was established through differential analysis, employing the random survival forest (RSF) algorithm and least absolute shrinkage and selection operator (LASSO) regression. The signature underwent comprehensive evaluations, including survival rate analysis, examination of clinical characteristics, and functional enrichment studies. Additionally, the LPRS was combined with other clinicopathological factors to create a prognostic nomogram specifically for LUAD patients. Integrated bioinformatics analyses identified KRT6A within the LPRS as a crucial prognostic marker, which was subsequently validated using clinical tissue specimens. This study systematically summarizes the critical role of LLPS in LUAD, screening and validating the significance of the key molecule KRT6A, ultimately contributing to more precise prognostic tools and targeted therapies for LUAD.

## 2 METHODS

### 2.1 Workflow of this STUDY

The study design flowchart is presented in Fig. (**1**). Initially, we summarized and extracted 3,585 LLPS-related genes from previous literature (Tables **S1-S2**). We explored the genomic variation landscape of these genes using The Cancer Genome Atlas Lung Adenocarcinoma (TCGA-LUAD) dataset. Subsequently, we established an LPRS using data from five bulk RNA sequencing cohorts (TCGA, GSE3141, GSE31210, GSE50081, GSE68465, and GSE72094) and a single-cell RNA sequencing cohort. We also conducted the functional analysis to explore the potential mechanisms by which LLPS influences LUAD prognosis. Finally, to ensure robust results, we validated the clinical significance of the key LLPS-related prognostic molecule, KRT6A, in a clinical cohort (ZZU cohort).

### 2.2 Variations in the Genetic Landscape of LLPS-Related Genes in LUAD

**Fig. (2):**
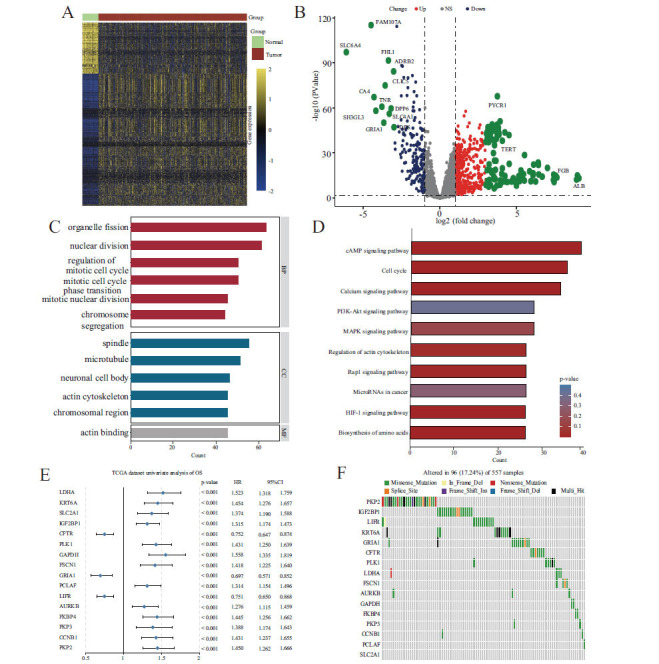
The variation and prognostic value of LLPS in LUAD. (**A**) Heatmap of the LLPS-related DEGs between LUAD and normal tissues. (**B**) Volcano plot of the LLPS-related DEGs. (**C**) GO enrichment analyses based on the DEGs. (**D**) KEGG enrichment analyses based on the DEGs. (**E**) Univariate Cox analysis of OS in LUAD. (**F**) CNV values of LLPS prognosis-related genes in the TCGA cohort.

By analyzing the TCGA-LIHC dataset, we identified 765 genes that displayed significant changes in expression levels (P-value and FDR < 0.05, and log2FC > 1), with 552 genes being upregulated and 213 genes downregulated in LUAD (Fig. **2A** and **B** and Table **S3**). KEGG and GO enrichment analyses revealed that these genes are involved in crucial biological processes, including nuclear division, organelle fission, cell cycle progression, and the cAMP signaling pathway (Fig. **2C** and **D**). An initial univariate Cox regression analysis identified 293 genes with expression levels significantly associated with overall survival (OS) at a significance level of less than 0.05. Further refinement using the bootstrapping technique identified 53 genes with consistent prognostic correlations across more than 900 iterations. Utilizing a minimal depth (MD)-based approach within the RSF methodology, we focused on the most crucial features for prognosis, repeating the RSF analysis 1,000 times to ultimately concentrate on 16 genes with the highest concordance index (C-index) values for further exploration. Fig. (**2E**) provides a graphical representation of the univariate Cox proportional hazards analysis for these 16 genes in the TCGA-LUAD dataset. Moreover, an analysis of copy number variation (CNV) status revealed recurrent genetic modifications in these genes, predominantly copy number amplifications, with LIFR showing the most frequent amplification in LUAD (Fig. **S1A-B**). The chromosomal positions of these 16 genes are illustrated in Fig. (**S1C**). Somatic mutation examination indicated a significant mutation prevalence of 17.2% (96/557) among these genes in LUAD patients (Fig. **2F**). Collectively, these observations suggest that LLPS-related genes exhibit widespread deregulation in LUAD and hold significant prognostic potential.

### 2.3 Computation of LPRS and Evaluation of its Clinical Relevance and Pathway Characteristics

**Fig. (3):**
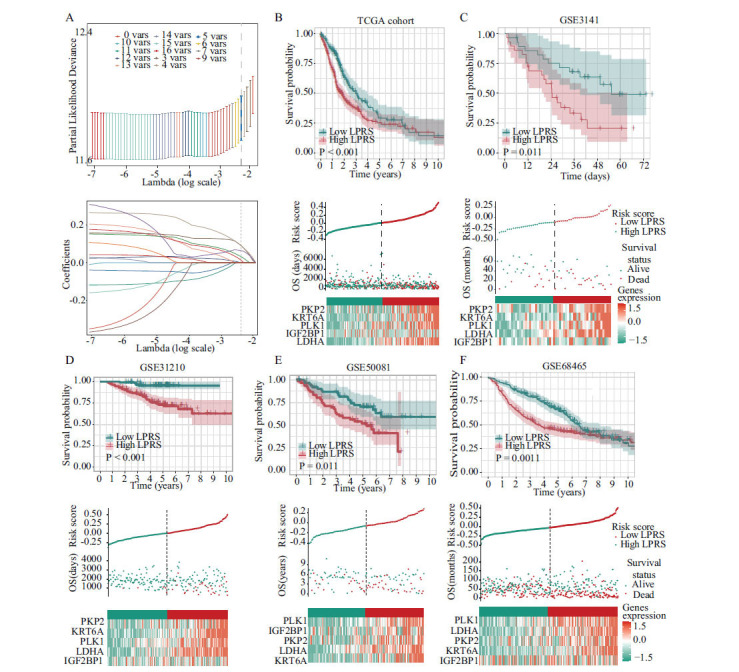
Construction and validation of the LPRS signature. (**A**) Selection of the optimal parameter (lambda) in the LASSO model. LASSO coefficients of the 5 LLPS-prognosis related genes in TCGA cohort. (**B**) Overall survival analysis for high-LPRS and low-LPRS groups in the training (TCGA) cohort. (**C**-**F**) Overall survival analysis for high-LPRS and low-LPRS groups in the validation (GSE3141, GSE31210, GSE50081, and GSE68465) cohort, respectively.

**Fig. (4):**
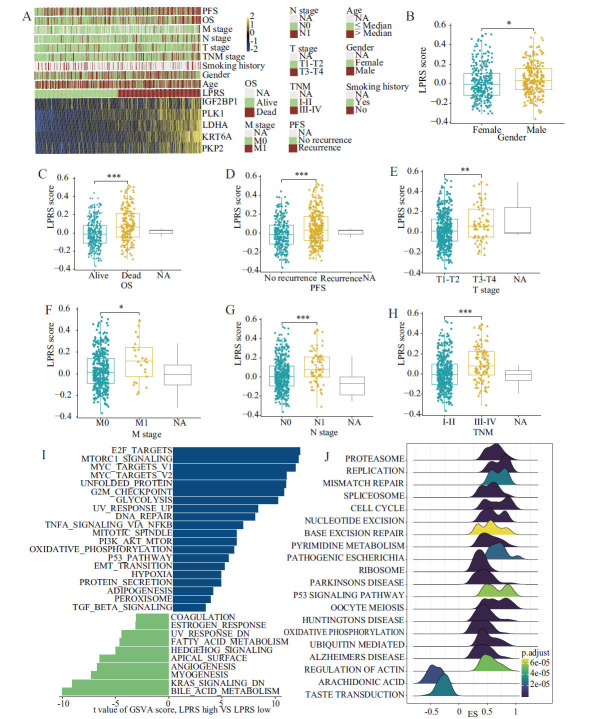
LPRS clinical value and pathway characteristic assessment. (**A**) Heatmap of five LLPS prognosis-related genes expression and corresponding clinicopathological features of low- and high-LPRS group. (**B-H**) The relationships between the LPRS and clinical characteristics, including gender, OS, PFS, T stage, M stage, N stage, and TNM stage. (**I**) GSVA enrichment results based on the TCGA database. (**J**) GSEA enrichment results based on the TCGA database.

To evaluate the prognostic relevance of LLPS-related genes in patients with LUAD, we calculated the LLPS-related prognostic risk score (LPRS) using the genes IGF2BP1, KRT6A, LDHA, PKP2, and PLK1. This score was generated *via* a LASSO Cox regression model, adhering to the minimum criterion (Fig. **3A**). The formula for LPRS calculation is LPRS = (0.079 × LDHA expression) + (0.078 × KRT6A expression) + (0.001 × IGF2BP1 expression) + (0.061 × PLK1 expression) + (0.024 × PKP2 expression), applied across both TCGA-LUAD and validation cohorts (GSE3141, GSE31210, GSE50081, GSE68465, and GSE72094). After categorizing patients into high- and low-LPRS groups based on the median LPRS value, a significant association was observed between increased LPRS and reduced OS, as shown in (Fig. **3B**-**F** and Fig. **S2A**). Further analysis linking LPRS with clinical parameters indicated significant associations of higher LPRS with male gender, more advanced TNM stages, and poorer outcomes regarding OS, progression-free survival (PFS), and advanced T, N, and M stages (Fig. **4A**-**H**). By using Gene Set Variation Analysis (GSVA) and Gene Set Enrichment Analysis (GSEA) to compare biological pathways in high- and low-LPRS groups from TCGA data (Fig. **4I** and **4J**), it was found that LPRS has an impact on the cell cycle. This effect was observed in pathways, such as HALLMARK MYC TARGETS V1, HALLMARK MYC TARGETS V2, CELL CYCLE, and HALLMARK G2M CHECKPOINT (Figs. **4I**, **4G**, **S2B**).

### 2.4 Development and Evaluation of the Nomogram Survival Model

**Fig. (5):**
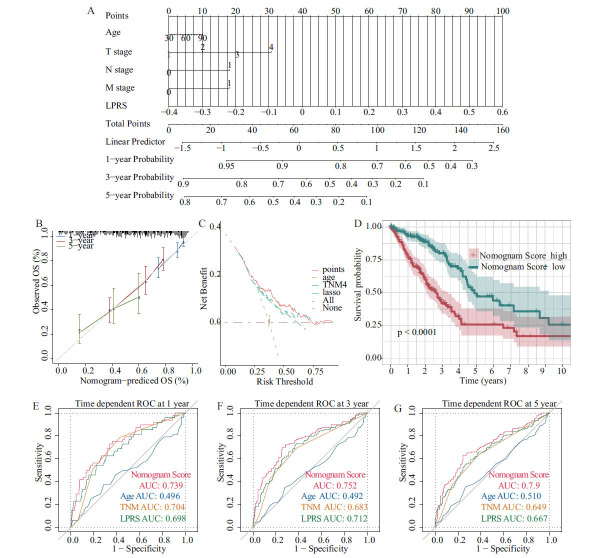
Establishment and assessment of the nomogram survival model. (**A**) A nomogram was established to predict the prognostic of LUAD patients. (**B**) Calibration plots showing the probability of 1-, 3-, and 5-year overall survival in the TCGA cohort. (**C**) DCA of a nomogram predicting overall survival. (**D**) Kaplan-Meier analyses for the two LUAD groups based on the nomogram score. (**E-G**) Receiver operator characteristic (ROC) analysis of nomogram predicting 1-, 3-, and 5-year overall survival in TCGA cohort.

Additional examination revealed that both univariate and multivariate Cox regression analyses validated the LPRS and TNM stages as independent prognostic factors for LUAD (Table **S4-S5**). A nomogram was created using data from The Cancer Genome Atlas (TCGA), incorporating both multivariate Cox and stepwise regression analyses, to forecast the 1-, 3-, and 5-year OS. This model incorporates critical predictors, such as age, TNM stage, and LPRS (Fig. **5A** and Table **S4**). Calibration curves confirmed the accuracy of the model's predictions for OS rates at 1, 3, and 5 years (Fig. **5B**). Decision curve analysis (DCA) highlighted the superior predictive capability of the nomogram compared to other clinical characteristics assessed in our study (Fig. **5C**). A significant difference in survival rates was observed between patients with high and low LPRS scores, as demonstrated by the nomogram in Fig. (**5D**). Furthermore, the area under the curve (AUC) metrics for the TCGA cohort highlighted the nomogram's outstanding predictive precision for 1-, 3-, and 5-year OS in LUAD patients (Fig. **5E**-**G**). Consequently, this prognostic nomogram for overall survival estimation is validated as both reliable and applicable for LUAD patients.

### 2.5 Analysis of the Tumor Microenvironment Using LPRS

**Fig. (6):**
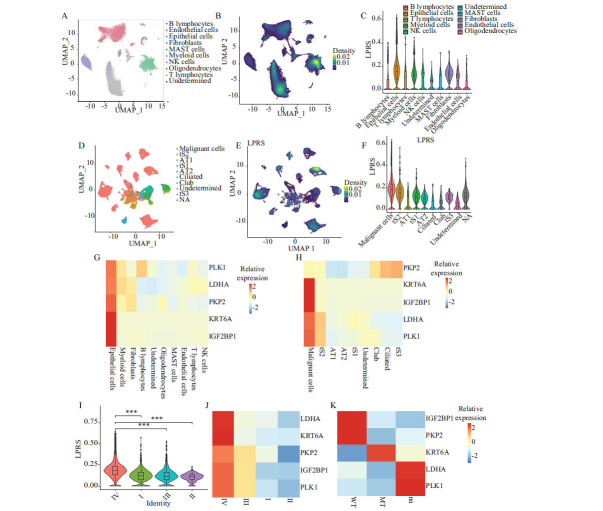
Analysis of the tumor microenvironment utilizing the LPRS signature. (**A**) UMAP plot visualization of cell subtypes across 11 LUAD patients. (**B)** UMAP plot depicting the density of LPRS. (**C**) Violin plots representing the LPRS value across different cell types. (**D**) UMAP plot illustrating the annotations of epithelial cells in LUAD patients. (**E**) UMAP plot showing the density of LPRS in the epithelial subgroup. (**F**) Violin plots of LPRS values in various epithelial subgroups. (**G** and **H**) Heatmaps displaying the distribution of genes from the 5-gene LPRS model within the cellular subtypes of the LUAD microenvironment. (**I**) Violin plots of LPRS values in malignant subgroups. (**J** and **K**) Heatmaps displaying the distribution of genes from the 5-gene LPRS model within clinical stages and EGFR mutation.

Single-cell RNA transcriptomics was employed to investigate the tumor microenvironment of LUAD and assess the distribution of the LPRS across various cellular types within the GSE131907 dataset. The analysis differentiated T lymphocytes and epithelial cells, revealing a significant divergence in LPRS scores compared to other cell types (Fig. **6A** and **B**). Violin plots clearly illustrated the discrepancies in LPRS scores across cell types, with epithelial cells exhibiting higher LPRS levels (Fig. **6C**). The substantial disparity in LPRS scores within epithelial cells necessitated further categorization (Fig. **6D**), demonstrating that cells with elevated LPRS predominantly belonged to malignant categories, and these malignant cells possessed significantly higher LPRS than other cell subtypes (Fig. **6E** and **F**). A heatmap further elucidated the expression patterns of five key genes (PLK1, LDHA, PKP2, KRT6A, and IGF2BP1) across different cell types, highlighting their pronounced expression within epithelial cells, particularly in malignant ones (Fig. **6G** and **H**). Moreover, the LPRS scores showed a notable increase in stage IV compared to stages I, II, and III, accompanied by a rise in the expression levels of the five genes in stage IV (Fig. **6I** and **J** and Table **S6**). Interestingly, KRT6A expression was higher in patients harboring EGFR mutations (Fig. **6K**). These findings underscore a significant association between LPRS and the prognosis and metastatic behavior of LUAD.

### 2.6 KRT6A as a Potential Oncogene in LUAD

**Fig. (7):**
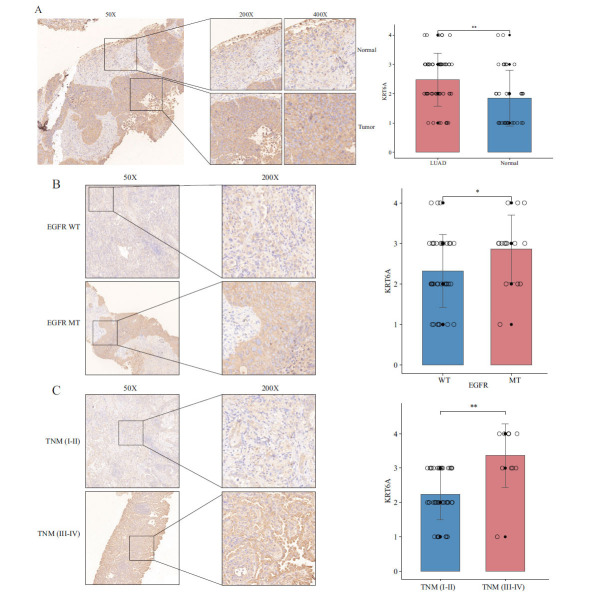
Immunohistochemical analysis of components of the LLPS-related prognosis signature in LUAD and normal lung tissue. (**A**) Level of expression of KRT6A in LUAD and adjacent normal lung tissue. (**B**) Relationship between KRT6A expression level and EGFR mutation. (**C**) Relationship between KRT6A expression level and TNM stage.

After a comprehensive examination of all findings, KRT6A emerged as a critical gene within the LPRS signature, warranting further experimental validation. The expression of KRT6A in LUAD and adjacent non-tumor lung tissues from the ZZU cohort was assessed using IHC experiments. Comparative analysis revealed a significant upregulation of KRT6A in tumor tissues (Fig. **7A** and Table **S7**). Tissues from advanced-stage (stage III and IV) patients exhibited markedly higher levels of KRT6A expression (Fig. **7C**), and tissues from patients with EGFR mutations demonstrated considerably higher KRT6A expression (Fig. **7B**). These findings suggest that KRT6A may function as an oncogene in LUAD.

## 3 RESULT AND DISCUSSION

Recent breakthroughs in understanding LLPS are revolutionizing our comprehension of tumor malignancy development [36-41]. In tumors, LLPS can influence tumor progression by affecting various biological processes, including transcriptional regulation, stress response, DNA replication, cellular redox homeostasis, and others [42, 43]. Consequently, this study aimed to investigate the clinical significance and molecular mechanisms of LLPS-related genes in LUAD. We first described the genomic variation landscape of LLPS-related genes in LUAD. Subsequently, we utilized comprehensive bioinformatics analyses to construct an LPRS that includes IGF2BP1, KRT6A, LDHA, PKP2, and PLK1.

Previous literature has systematically summarized the roles and potential mechanisms of IGF2BP1, MNX1-AS1, KRT6A, LDHA, and PKP2 in tumors, particularly in LUAD. IGF2BP1 may promote target gene expression by increasing mRNA stability and translation in an N6-methyladenosine (m6A)-dependent manner [44-46]. MNX1-AS1 induces IGF2BP1 phase separation, enhancing c-Myc and E2F1 signaling and triggering cell-cycle advancement to stimulate proliferation in NSCLC [47, 48]. Increased KRT6A expression in LUAD enhances lung cancer cell proliferation, migration, and colony formation through epithelial-mesenchymal transition (EMT) and cancer stem cell (CSC) transformation [49]. Xiao *et al.* noted in a bioinformatics study that high KRT6A expression was associated with poor prognosis in LUAD patients [50-52]. LDHA, a key enzyme in redox reactions, catalyzes the conversion of lactic acid to pyruvate in the glycolysis pathway, representing the end result of anaerobic glycolysis [53, 54]. Elevated LDHA expression may serve as a potential prognostic indicator for reduced survival in LUAD [54, 55]. PKP2 enhances cancer cell proliferation, mitosis, and movement by stimulating the EGFR signaling pathway in LUAD cells [56-59]. PLK1, a crucial regulator of cell division, is upregulated in lung cancer and other cancer types [60-64]. Elevated PLK1 levels are associated with decreased survival rates in cancer patients [63]. Blocking PLK1 activity prevents successful mitosis, inducing G2/M phase arrest and ultimately leading to apoptotic cell death. As G2/M is the most radiation-sensitive cell cycle phase, PLK1 inhibition also sensitizes cancer cells to irradiation [65]. Therefore, the LPRS constructed based on these five genes may hold significant importance for predicting LUAD prognosis and clinical progression.

The clinical significance and potential mechanisms of LPRS in LUAD were further evaluated. The analysis highlighted distinct survival differences between groups with high and low LPRS scores, with patients exhibiting elevated LPRS levels generally experiencing poorer clinical outcomes and survival rates compared to those with lower scores. Moreover, a prognostic nomogram was constructed, incorporating LPRS, TNM stages, and age. This model demonstrated high effectiveness in predicting survival outcomes, enhancing the accuracy of prognostic estimations in LUAD. Additionally, the role of LPRS in the LUAD tumor microenvironment was explored from a single-cell perspective. Notably, both the LPRS score and the expression levels of the five LLPS prognosis-related genes were significantly higher in malignant cells and cells with advanced TNM stages. Further investigation revealed that KRT6A was significantly overexpressed in EGFR-mutant LUAD cells, leading to the selection of KRT6A for further study.

Recent research indicates that LLPS contributes to the activation of signaling pathways associated with lung cancer, such as EGFR, anaplastic lymphoma kinase (ALK), and Kirsten rat sarcoma viral oncogene homolog (KRAS) [34, 66-69]. LLPS has been shown to promote EGFR recruitment to the CREB-binding protein (CBP)/p300 co-activator complex, enhancing the transcription of EGFR target genes involved in cell cycle progression, including cyclin D1 and B-Myb [70-72]. Additionally, LLPS of the non-POU domain-containing octamer-binding protein (NONO) has been demonstrated to increase EGFR recruitment to the cyclooxygenase-2 (COX-2) promoter, resulting in elevated COX-2 levels and promoting tumor progression [72, 36]. Targeting the dysregulation of LLPS in lung cancer cells may provide novel opportunities for developing improved therapies for this devastating disease. The formation of LLPS droplets can recruit and activate EGFR and downstream signaling molecules, leading to increased cell proliferation, survival, angiogenesis, invasion, and radioresistance [73, 74]. Our findings suggest that the expression of the LLPS-associated gene KRT6A correlates with EGFR mutations, potentially impacting downstream EGFR signaling pathways. Modulation of KRT6A could potentially help address resistance to EGFR tyrosine kinase inhibitors (EGFR-TKIs). In conclusion, the identification of LLPS-related prognostic signatures provides valuable insights into LUAD, potentially improving clinical outcome prediction and treatment selection.

## CONCLUSION

In this study, we developed a novel LPRS model that accurately predicts the clinical prognosis of LUAD. LLPS may significantly influence LUAD prognosis by modulating its metastatic propensity. Furthermore, accumulating evidence suggests that KRT6A is a pivotal factor in the LLPS-mediated malignant progression of LUAD.

## Figures and Tables

**Fig. (1):**
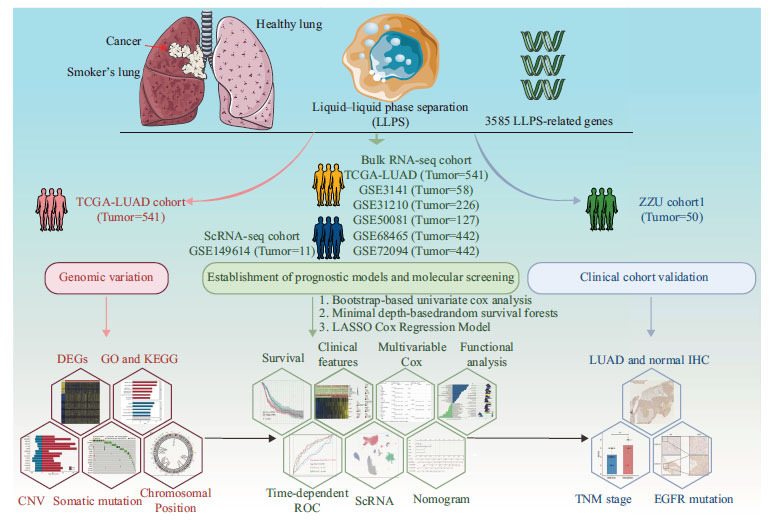
Flowchart for comprehensive analysis of LLPS in LUAD.

## Data Availability

The data and supportive information are available within the article.

## LIST OF ABBREVIATIONS

GEO: Gene Expression Omnibus
LLPS: Liquid-liquid Phase Separation
LPRS: LLPR-related Prognosis Score
LUAD: Lung Adenocarcinoma
NSCLC: Non-small-cell Lung Cancer
RSF: Random Survival Forest
TCGA: The Cancer Genome Atlas

## STANDARDS OF REPORTING

Strobe guidelines were followed.
