# Supplementary material for: Liquid-Liquid Phase Separation in the Prognosis of Lung Adenocarcinoma: An Integrated Analysis
Source: Curr Cancer Drug Targets. 2024 Nov 6;25(4):323–34. doi: 10.2174/0115680096345676241001081051 (PMC12174900; doi:10.2174/0115680096345676241001081051)
Supplement: Supplementary file 1 [file CCDT-25-4-323_SD1.pdf]

Supplementary Material

Liquid-Liquid Phase Separation in the Prognosis of Lung Adenocarcinoma: An Integrated Analysis

Qilong Wang[1], Nannan Sun[2], Jianhao Li[3], Fengxiang Huang[1] and Zhao Zhang[1,*]

[1]Department of Respiration, The First Affiliated Hospital of Zhengzhou University, Zhengzhou, Henan, People's Republic of China; [2]Department of Hematology, The First Affiliated Hospital of Zhengzhou University, Zhengzhou, Henan, People's Republic of China; [3]Precision Medicine Center, The First Affiliated Hospital of Zhengzhou University, Zhengzhou, Henan, People's Republic of China

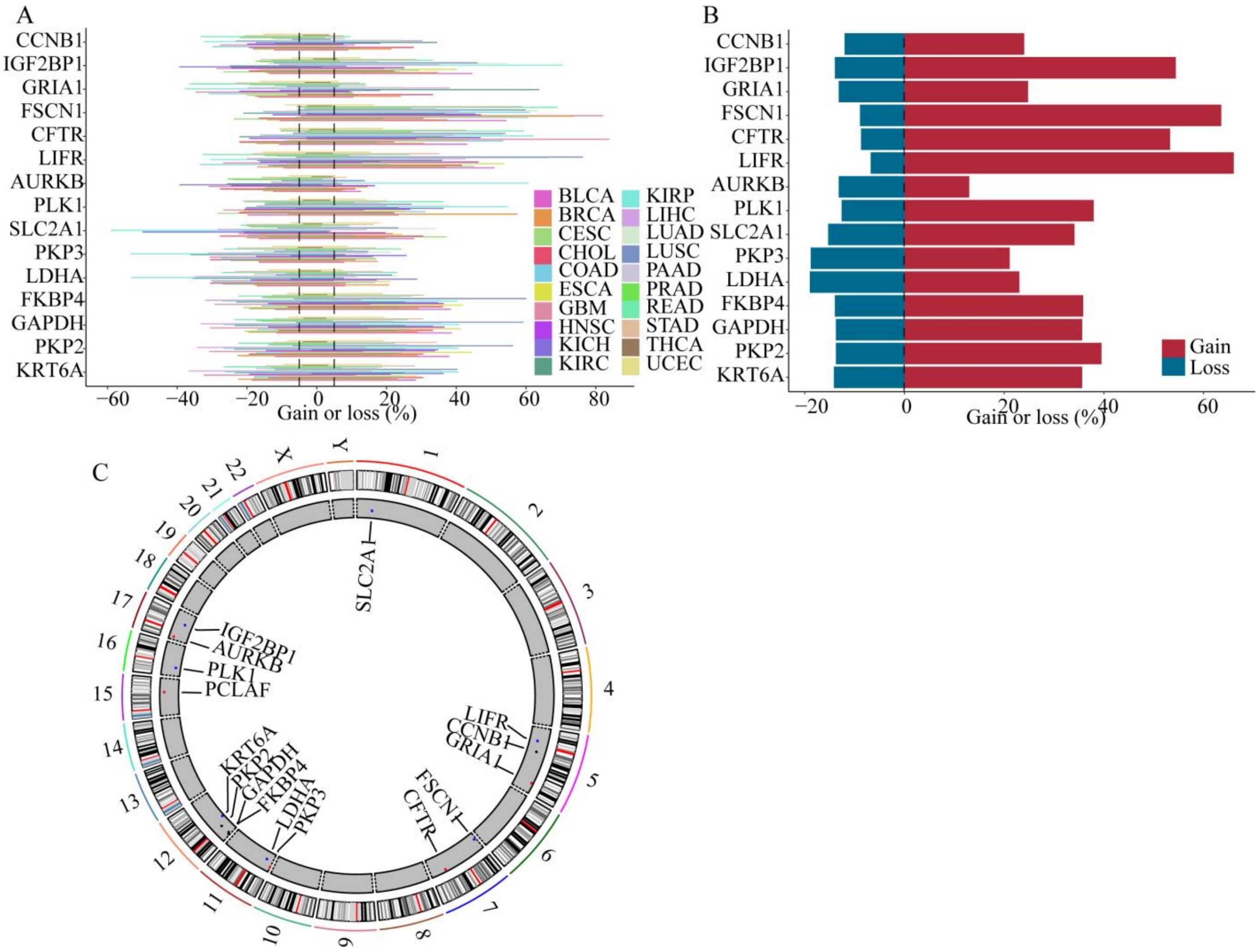

Fig. (S1). Variation and chromosomal distribution of LLPS prognosis-related genes in LUAD. (**A**) CNV values of LLPS prognosis-related genes across various cancers as per TCGA database. (**B**) Somatic mutations in LLPS prognosis-related genes according to TCGA data. (**C**) Chromosomal distribution of LLPS prognosis-related genes.

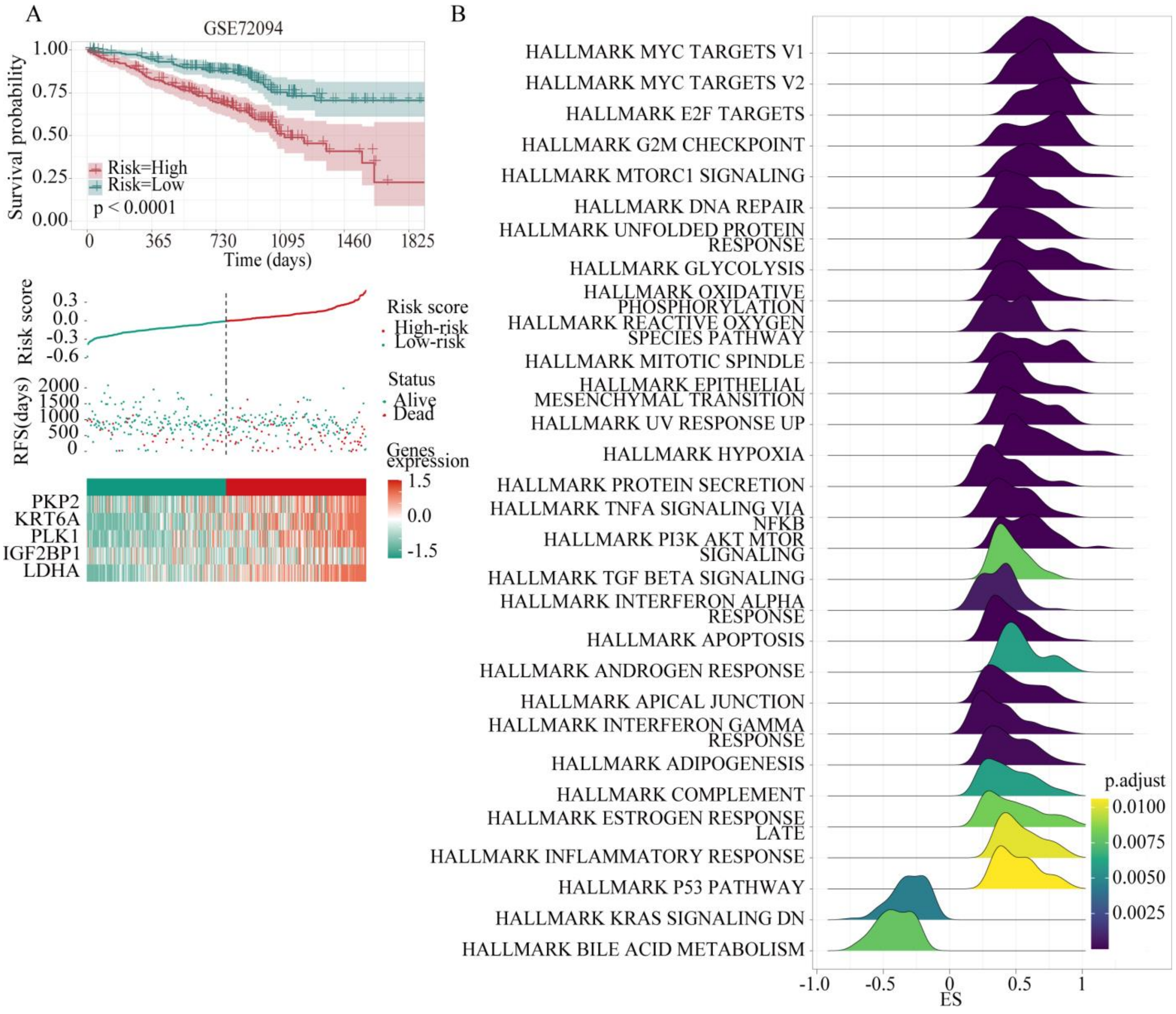

Fig. (S2). Prognostic and pathway characteristics of LPRS. (A) Univariate and multivariate Cox analyses of clinicopathological characteristics and LPRS in the TCGA cohort. (B) GSEA comparing high- and low-LPRS patients.
